# Identification of Molecular Markers Related to Immune Infiltration in Patients with Severe Asthma: A Comprehensive Bioinformatics Analysis Based on the Human Bronchial Epithelial Transcriptome

**DOI:** 10.1155/2022/8906064

**Published:** 2022-11-03

**Authors:** Yong Jiang, Qian Yan, Miaofen Zhang, Xueying Lin, Chenwen Peng, Hui-ting Huang, Gang Liao, Qiong Liu, Huili Liao, Shao-feng Zhan, Xiaohong Liu, Xiufang Huang

**Affiliations:** ^1^Shenzhen Hospital of Integrated Traditional Chinese and Western Medicine, Shenzhen, China; ^2^The First Affiliated Hospital of Guangzhou University of Chinese Medicine, Guangzhou, China; ^3^Guangzhou University of Chinese Medicine, Guangzhou, China; ^4^Lingnan Medical Research Center of Guangzhou University of Chinese Medicine, Guangzhou, China

## Abstract

**Background:**

Severe asthma (SA), a heterogeneous inflammatory disease characterized by immune cell infiltration, is particularly difficult to treat and manage. The airway epithelium is an important tissue in regulating innate and adaptive immunity, and targeting airway epithelial cell may contribute to improving the efficacy of asthma therapy.

**Methods:**

Bioinformatics methods were utilized to identify the hub genes and signaling pathways involved in SA. Experiments were performed to determine whether these hub genes and signaling pathways were affected by the differences in immune cell infiltration.

**Results:**

The weighted gene coexpression network analysis identified 14 coexpression modules, among which the blue and salmon modules exhibited the strongest associations with SA. The blue module was mainly enriched in actomyosin structure organization and was associated with regulating stem cell pluripotency signaling pathways. The salmon module was mainly involved in cornification, skin development, and glycosphingolipid biosynthesis-lacto and neolacto series. The protein-protein interaction network and module analysis identified 11 hub genes in the key modules. The CIBERSORTx algorithm revealed statistically significant differences in CD8+ T cells (*P* = 0.013), T follicular helper cells (*P* = 0.002), resting mast cells (*P* = 0.004), and neutrophils (*P* = 0.002) between patients with SA and mild-moderate asthma patients. Pearson's correlation analysis identified 11 genes that were significantly associated with a variety of immune cells. We further predicted the utility of some potential drugs and validated our results in external datasets.

**Conclusion:**

Our results may help provide a better understanding of the relationship between the airway epithelial transcriptome and clinical data of SA. And this study will help to guide the development of SA-targeted molecular therapy.

## 1. Introduction

Severe asthma (SA) is a chronic respiratory disease that worsens with a reduction in treatment with high-dose inhaled corticosteroids and long-acting *β*_2_ agonist (ICS-LABA), or symptoms persist despite treatment with high-dose therapy targeting the causative agent, as defined by the Global Initiative for Asthma (GINA) 2022 [1]. Unfortunately, approximately 300 million people suffer from asthma worldwide, of which 3-10% are diagnosed with SA [1, 2]. According to statistics, the death rate of patients with asthma has not significantly decreased in the past 30 years, indicating that asthma/SA has not been well controlled [3]. Unsurprisingly, SA has imposed substantial physical and economic burdens on patients and society [4–6].

SA is clinically characterized by chronic and persistent inflammation and airway hyperreactivity (AHR). The inflammatory phenotype of SA may be dominated by type 2 inflammation, neutrophilic inflammation, and mixed inflammation, which is possibly regulated by T lymphocytes, neutrophils, eosinophils, and airway epithelial cells [7]. Airway epithelial cells, frontline guardians of the body's defense system, play important roles in regulating innate and adaptive immunity [8]. Proinflammatory cytokines may lead to the degradation of the rhythmic circadian repressor REV-ERB*α* in airway epithelial cells, resulting in a rhythmic inflammatory response that may be associated with asthma [9]. In addition, the mechanism underlying immune cell infiltration in patients with SA has attracted increasing attention from researchers. For example, type 2 innate lymphoid cells (ILC2s) induce eosinophilic infiltration and AHR, which are associated with the onset of type 2 asthma [10]. Therefore, approaches targeting airway epithelial cells and mechanisms of immune cell infiltration may reveal important strategies to control and improve SA.

**Figure 1:**
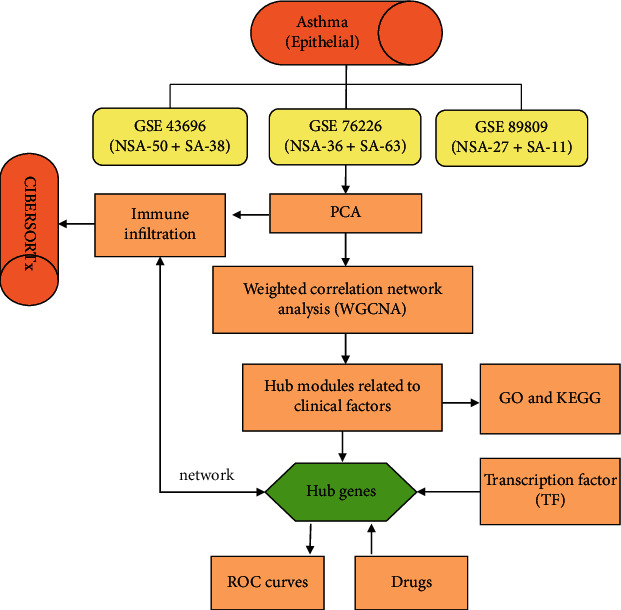
The flowchart of the systemic approach to identify potential targets and therapies for SA. SA: severe asthma; NSA: mild and moderate asthma; PCA: principal component analysis; GO: Gene Ontology; KEGG: Kyoto Encyclopedia of Genes and Genomes.

Weighted gene coexpression network analysis (WGCNA) is a system biology method that has been used to find the important modules with highly correlated genes in microarray samples. WGCNA can identify therapeutic targets for SA by linking genes in modules with external sample features [11]. CIBERSORTx, a commonly used technical method in the field of immunology, uses a deconvolution algorithm to evaluate the infiltration of immune cells in tissues [12]. The aim of this study was to identify novel mechanisms underlying immune cell infiltration and biomarkers of SA using transcriptomic data from airway epithelial cells. The workflow of the bioinformatics analysis is shown in Figure 1.

## 2. Materials and Methods

### 2.1. Dataset Preparation

We obtained the genomic mRNA profiles and corresponding clinical data for patients with SA from the NCBI Gene Expression Omnibus (GEO, https://www.ncbi.nlm.nih.gov/geo/) database [13] with the following criteria: (“asthma” [MeSH Terms] OR asthma [All Fields]) AND (“gse” [Filter] AND “Homo sapiens” [Organism] AND “Expression profiling by array” [Filter]). In addition, the dataset must contain clinical information for patients with varying levels of asthma severity, and the samples must originate from bronchial epithelial cells. We also excluded childhood asthma and datasets with fewer than 30 samples. Hence, the GSE43696, GSE76226, and GSE89809 datasets were selected in subsequent studies. Because the data in this study were derived from public databases, the approval of the ethics committee was not needed.

### 2.2. Removal of the Batch Effect

The ComBat function was used to normalize the datasets from different batches for further analysis, and the R package “SVA” was used to eliminate the batch effect [14]. Then, the principal component analysis (PCA) was performed to evaluate whether the batch effect was removed.

### 2.3. Coexpression Network Construction and Disease-Specific Module Identification

The top 5000 genes in three datasets (sorted by adjusted *P* value) and the “WGCNA” package in R were used to construct WGCNA network and determine the coexpression modules related to SA [11]. First, the goodSampleGenes function in WGCNA was used to find missing values, and we performed a cluster analysis on 225 samples to identify free samples. An *R*-square value of 0.85 was set as the screening criterion, scatter plots of the fitting index, average connectivity, and soft threshold (power) were constructed to determine the best scale-free network, and the best weighting coefficient *β* was selected. The dynamic hybrid cutting method was used to identify modules, and similar modules were clustered and merged. The following parameters were set: minModuleSize = 60, mergeCutHeight = 0.3, deepSplit = 2, and verbose = 3. Finally, we performed Pearson's correlation analysis between modules and clinical features. The characteristic genes associated with SA were selected for subsequent analysis, and *P* < 0.05 was set as the filtered criterion.

### 2.4. Functional Enrichment Analyses

The Gene Ontology (GO) enrichment analysis included biological processes (BPs), cellular components (CCs), and molecular functions (MFs). The R package “clusterProfiler” was used to perform GO and Kyoto Encyclopedia of Genes and Genomes (KEGG) enrichment analyses of genes associated with significant modules and further explore the molecular mechanism associated with SA [15]. GO terms or KEGG terms with the criterion of *P* < 0.05 were considered significantly enriched.

### 2.5. Protein-Protein Interaction (PPI) Network Construction and Module Analysis

The protein-protein interaction (PPI) network was constructed using the STRING database (version 11.0, https://www.string-db.org/) to show the correlations of various protein targets in important modules. Molecular Complex Detection (MCODE) analysis in Cytoscape software 3.8.2 (https://www.cytoscape.org/) was performed to screen central nodes in the key modules, and the screening criteria were set as follows: degree cutoff = 2, node score cutoff = 0.2, k − core = 5, and max depth = 100 [16]. The “degree” value in the “CytoHubba” plugin in Cytoscape was used to screen key targets in the module. Finally, the Network Analyst (version 3.0, https://www.networkanalyst.ca/) online website was used to visualize the key modules [17].

### 2.6. Evaluation of the Abundance of Immune Cell Infiltration

We uploaded the normalized gene expression profiles from the GSE43696, GSE76226, and GSE89809 datasets to CIBERSORTx (https://cibersortx.stanford.edu/) [12]. CIBERSORTx used the deconvolution algorithm to calculate the abundances of immune cells in each sample based on the expression levels of 22 immune cell-related genes and the matrix of gene expression [18].

### 2.7. Analysis of the Associations between Hub Genes and Immune Cells

The “ggstatsplot” R package (https://CRAN.R-project.org/package=ggstatsplot) was used to perform Pearson's correlation analysis between the identified genes and the levels of infiltrating immune cells with R and to further analyze the immune mechanism involved in the development of SA.

### 2.8. Analysis of the Transcription Factor-Gene Interaction Network

Network Analyst (version 3.0), a visualization platform, was used to construct the interaction network between TFs and genes [17]. The basic data for the TF-gene network were obtained from the ENCODE database (https://www.encodeproject.org/) and visualized using Cytoscape software 3.8.2 [16].

### 2.9. Identification of Candidate Drug-Targets

The ToppGene Database (https://toppgene.cchmc.org/help/help.jsp) was utilized to identify the potential pharmacological drugs for SA [19]. The list of 11 hub genes was used to capture drug-gene interactions by using the ToppGene Database.

### 2.10. Receiver Operating Characteristics (ROC) Analysis

A ROC curve analysis was conducted for each hub gene in patients with mild asthma (MA) and SA. The area under the curve (AUC) was calculated to evaluate the diagnostic accuracy of the six hub genes for SA by using the “pROC” R package [20].

### 2.11. Statistical Analysis

All statistical analyses were performed using R (version: 4.1.0, https://www.r-project.org/). The Wilcoxon test was used to judge whether the two groups had differences in immune cell infiltration. Pearson's correlation coefficients were calculated to determine the associations between modules and clinical factors, and Spearman's correlation analysis was performed to evaluate the associations between candidate genes and immune cells. The Bonferroni method was used to correct the *P* values, and an adjusted *P* value < 0.05 was set as the filtering criterion.

## 3. Results

### 3.1. Removal of the Batch Effect by Cross-Platform Normalization

**Figure 2:**
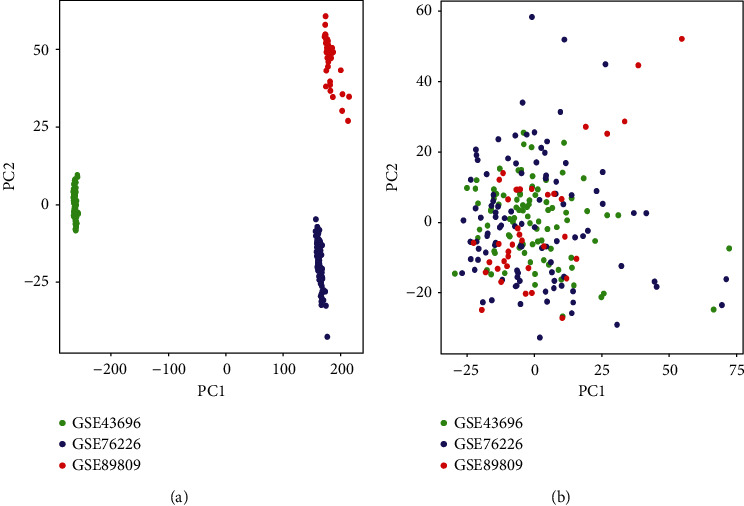
The principal component analysis (PCA) of the GSE43696, GSE76226, and GSE89809 datasets in severe asthma. (a) Before removing batch effects. (b) After removing batch effects. The colors represented samples from three different datasets, respectively.

GSE43696 was obtained from the GPL6480 platform (Affymetrix Human Genome U133 Plus 2.0 Array), including 50 patients with moderate asthma and 39 patients with SA. GSE76226 was derived from the GPL13158 platform and included 36 patients with moderate asthma and 63 patients with SA. GSE89809 originated from the GPL13158 platform and comprised 28 patients with mild asthma, 13 patients with moderate asthma, and 11 patients with SA. The normalized results of the PCA based on the scatter plot showed that the batch effects generated by the three datasets corresponding to different platforms were significantly removed (Figure 2).

### 3.2. Identification of Gene Coexpression Modules for Each Subgroup

**Figure 3:**
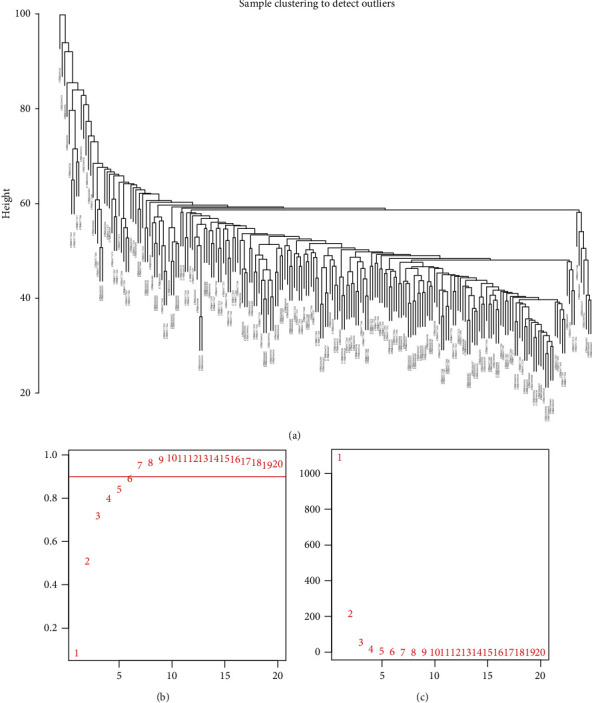
The construction of weight gene coexpression network. (a) Sample cluster analyses. (b) Scatter plot of fitting index and the value of soft threshold (power). (c) Scatter plot of the mean connectivity and the value of soft threshold (power). The abscissa is the value of power, and the ordinate is the scare-free fit index (b) and mean connectivity (c). The higher the value of the ordinate, the more the network conforms to the scale-free feature.

**Figure 4:**
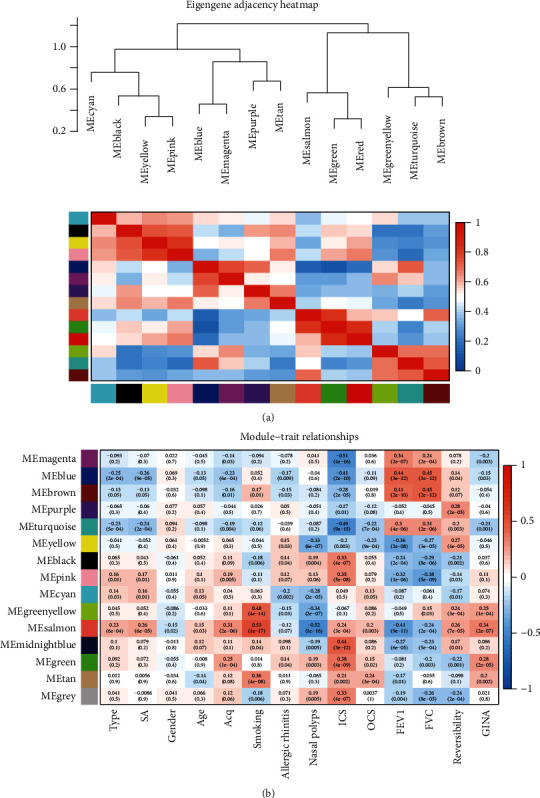
Identification of the modules related to severe asthma. (a) Eigengene dendrogram showed the correlation between modules. (b) Heatmap showed the association between genes in modules with clinical factors. SA: severe asthma; ACQ: asthma control questionnaire; ICS: inhaled corticosteroid; OCS: oral corticosteroid; FEV1: forced expiratory volume in the first second; FVC: forced vital capacity; GINA: Global Initiative for Asthma.

As a method to identify SA-related coexpression modules, the R package “WGCNA” was used to construct signed networks based on the expression levels of 11105 genes in the three datasets. First, the cluster analysis of samples did not reveal free samples. The correlation coefficient was greater than 0.85 when *β* equaled to 6 (Figure 3). The coexpression network was a scale-free network, and 14 coexpression modules were identified (Figure 4(a)). The blue module (correlation coefficient = −0.26, *P* = 9e − 05) and salmon module (correlation coefficient = 0.26, *P* = 6e − 05) had the strongest correlations with SA. The blue module was negatively correlated with the asthma control questionnaire (ACQ), allergic rhinitis (AR), inhaled corticosteroid (ICS) dose, oral corticosteroid (OCS) dose, and Global Initiative for Asthma (GINA) control. The blue module positively correlated with the forced expiratory volume in the first second (FEV_1_), forced vital capacity (FVC), and reversibility. The salmon module was negatively correlated with gender, AR, nasal polyps (NP), FEV_1_, and FVC, while the correlation between the salmon module and age, ACQ score, smoking, ICS, OCS, reversibility, and GINA showed a positive relationship (Figure 4(b)). Each module was associated with specific symptoms of asthma, except for the genes in the gray modules that had no clinical significance.

### 3.3. Functional Enrichment Analysis

**Figure 5:**
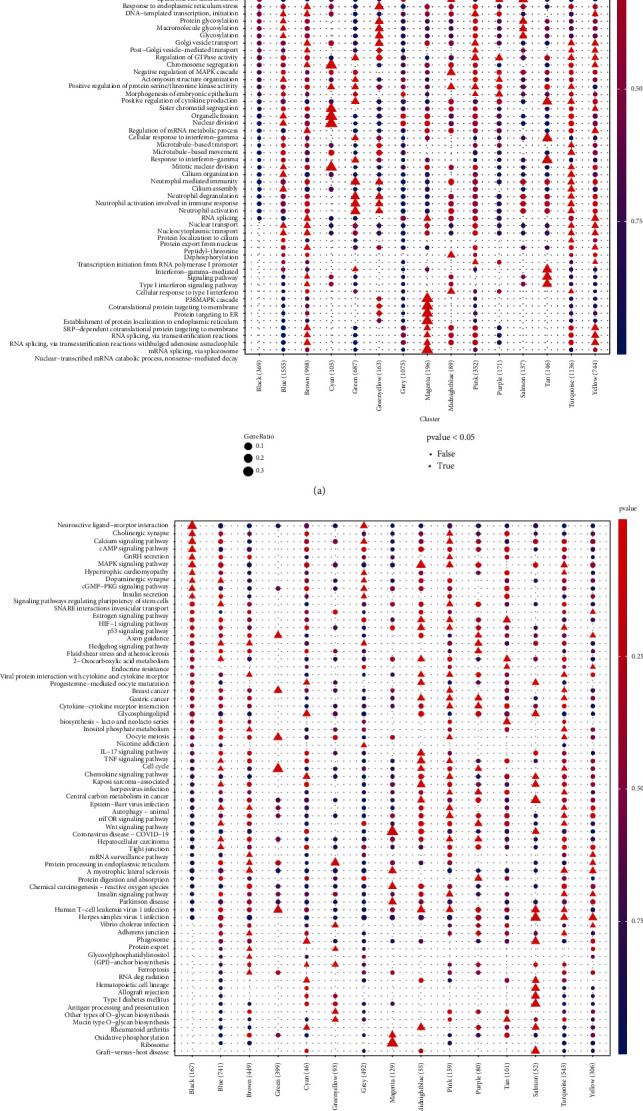
Biological process (a) and Kyoto Encyclopedia of Genes and Genomes (b) pathway enrichment analyses for genes in all modules.

We performed GO (BP) and KEGG enrichment analyses of 14 coexpression modules. The blue module was mainly enriched in actomyosin structure organization, muscle cell differentiation, cilium organization, striated muscle cell differentiation, sodium ion transmembrane transport and positive regulation of the catabolic process, and signaling pathways involved in regulating pluripotency of stem cells. The salmon module was mainly enriched in cornification, skin development, and various metabolic processes, as well as glycosphingolipid biosynthesis-lacto and neolacto series, mucin-type O-glycan biosynthesis, and the estrogen signaling pathway. The top five pathways in all modules were ribosome, coronavirus disease-COVID-19, antigen processing and presentation, graft-versus-host disease, and allograft rejection, as ranked by adjusted *P* values, all of which were associated with the regulation of the host's immune system (Figure 5).

### 3.4. PPI Network Construction and Module Analysis

**Figure 6:**
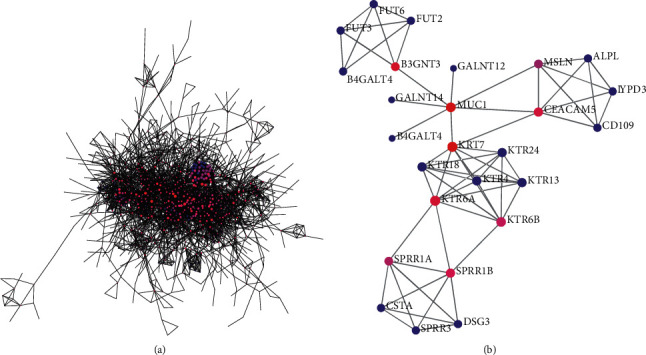
The construction of protein-protein interaction network in blue (a) and salmon (b) modules.

**Figure 7:**
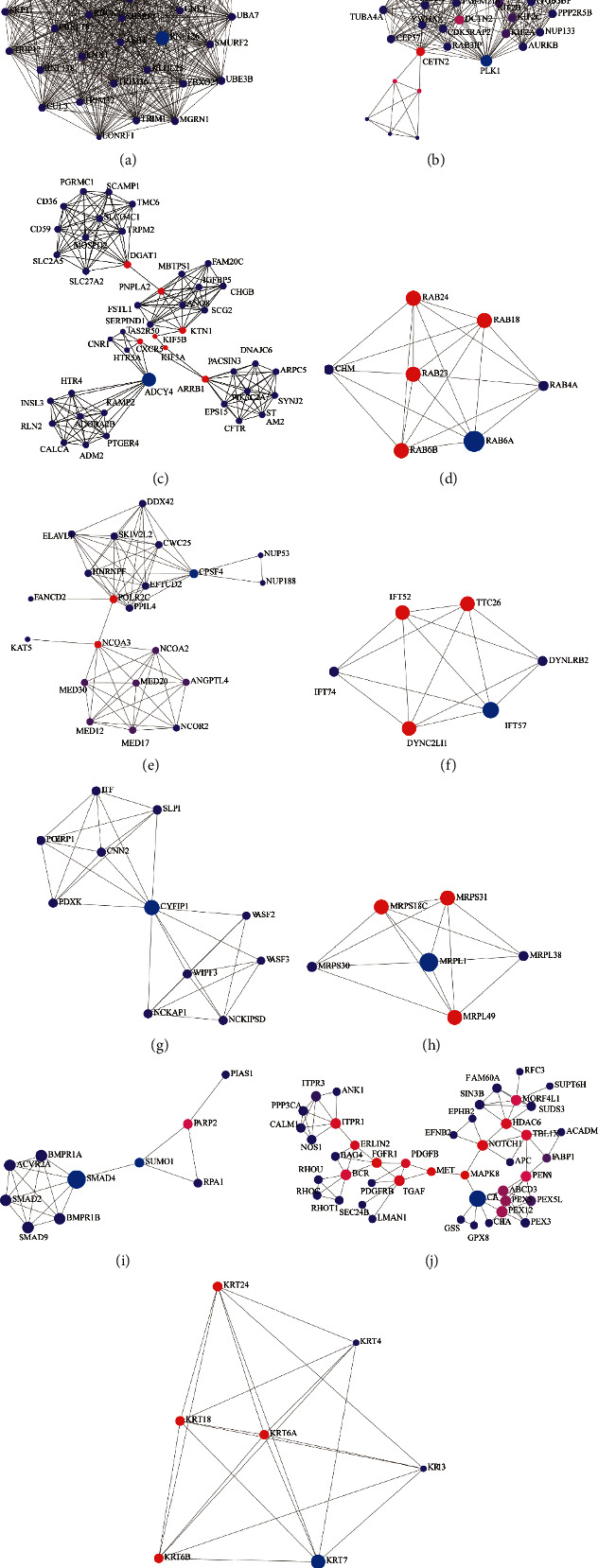
Module analysis of the blue and salmon modules identified by weighted gene coexpression network analysis. (a–j) The first ten modules showed the key modules in the blue module, and the last (k) was the key module in the salmon module.

The genes in the blue and salmon modules were uploaded to the STRING database for the PPI network analysis. The blue module contained 1731 nodes and 4228 edges, and the salmon module contained 144 nodes and 70 edges with a combined score greater than 0.7 and deletion of discrete targets (Figure 6). Then, we used the MCODE function in Cytoscape software 3.8.2 to analyze the key modules, and CytoHubba was used to screen the critical nodes with the highest degree values in the module. The blue module contained 10 modules, among which the 10 hub genes were RNF126, PLK1, ADCY4, RAB6A, CPSF4, IFT57, CYFIP1, MRPL1, SMAD4, and CAT. The salmon module contained a module that met the requirements, and the central gene was KRT18 (Figure 7).

### 3.5. Analysis of the Degree of Immune Cell Infiltration

**Figure 8:**
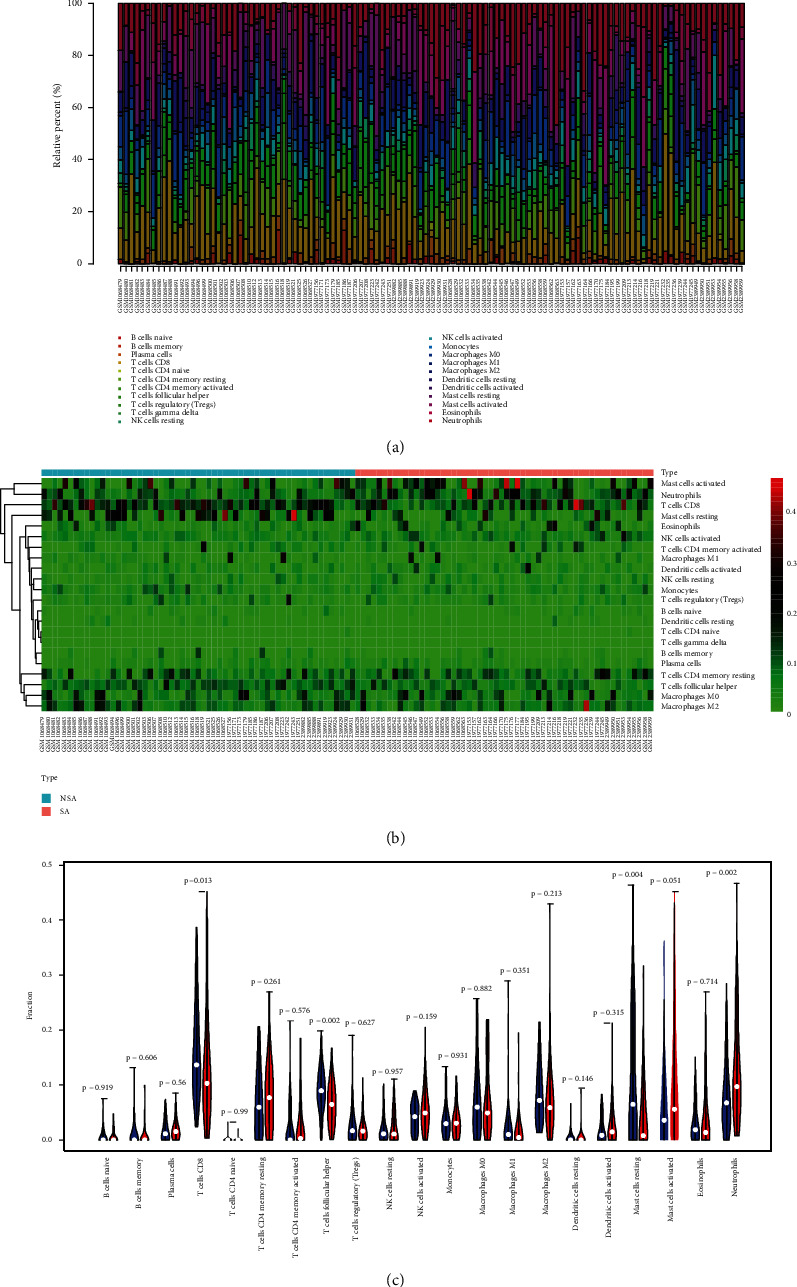
Evaluation and visualization of immune cell infiltration in the three datasets using CIBERSORTx. (a) Histogram of the fraction of 21 types of immunity in each sample. (b) Heatmap of the contents of 21 types of immune cells in each sample. (c) Violin plot of the contents of 21 types of immune cells in each sample.

CIBERSORTx was used to calculate the deconvoluted *P* value for each sample to provide a measure of confidence in the result, and *P* < 0.05 was considered statistically significant. We filtered out the immune cells that were not abundant in the samples (gamma delta T cells). The results of the analysis of differential infiltration analysis of the 21 types of immune cells showed that the differences in the ratios of CD8 T cells (*P* = 0.013), follicular helper T cells (*P* = 0.002), resting mast cells (*P* = 0.004), and neutrophils (*P* = 0.002) between patients with SA and mild-moderate asthma were statistically significant. This result implied that the contents of CD8 T cells, follicular helper T cells, and resting mast cells in SA samples were lower than those in mild-moderate asthma samples, and greater neutrophil infiltration was observed in samples from patients with SA (Figure 8).

### 3.6. Analysis of the Correlations between Hub Genes and Immune Cells

**Figure 9:**
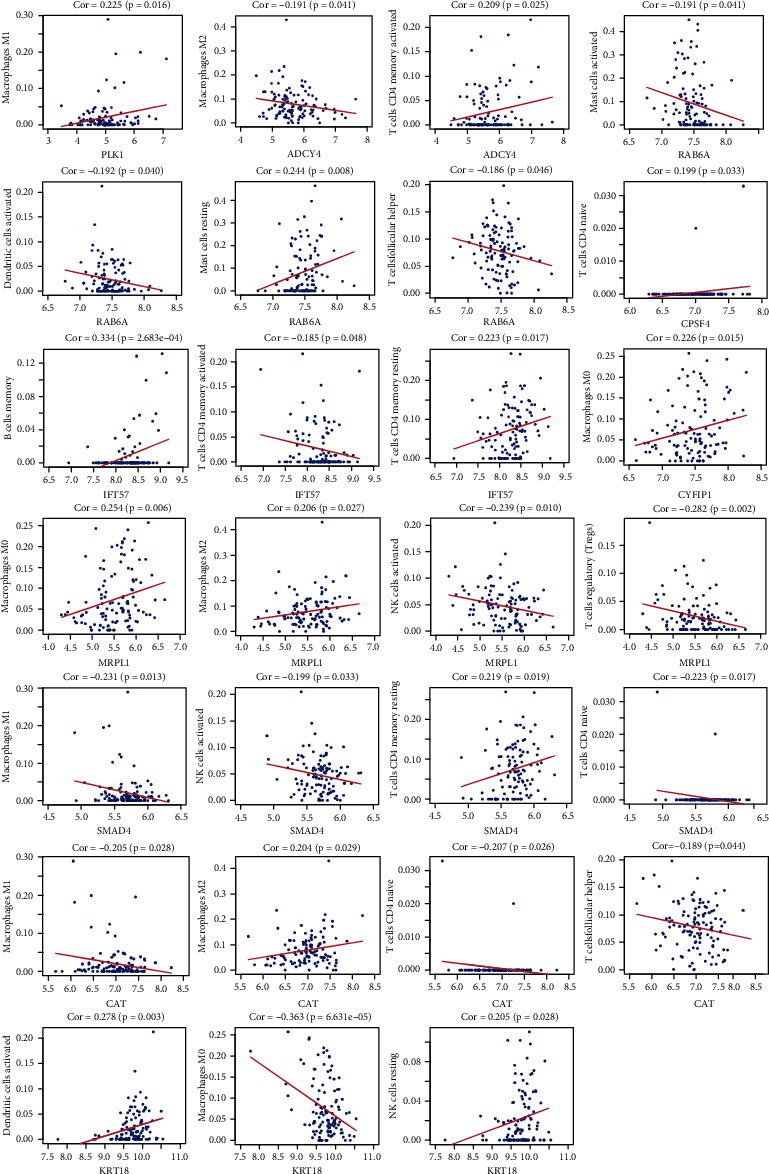
Pearson correlation analyses between 21 immune cells and hub genes.

Pearson's correlation analysis was performed to calculate the correlation coefficients between 11 hub genes and 21 immune cells. M0 macrophages were positively correlated with CYFIP1 and MRPL1 and negatively correlated with KRT18. M1 macrophages were positively correlated with PLK1 and negatively correlated with SMAD4 and CAT. M2 macrophages were negatively correlated with ADCY4 and positively correlated with MRPL1 and CAT. Activated CD4 memory T cells were positively correlated with ADCY4 and negatively correlated with IFT57. Resting memory CD4 T cells were positively correlated with IFT57 and SMAD4. T cell follicular helper was negatively correlated with RAB6A and CAT. Naive CD4 T cells were positively correlated with CPSF4 and negatively correlated with SMAD4 and CAT. Tregs was negatively correlated with MRPL1. Memory B cells were positively correlated with IFT57. Activated NK cell activation was negatively correlated with MRPL1 and SMAD4. Resting NK cells were positively correlated with KRT18. Resting mast cells were positively correlated with RAB6A, and mast cell activation had a negative correlation with RAB6A. Activated dendritic cells were negatively correlated with RAB6A and positively correlated with KRT18. Statistically significant correlations were not observed between RNF126 and the 21 types of immune cells (Figure 9).

### 3.7. Transcription Factor-Hub Gene Coregulation Network

**Figure 10:**
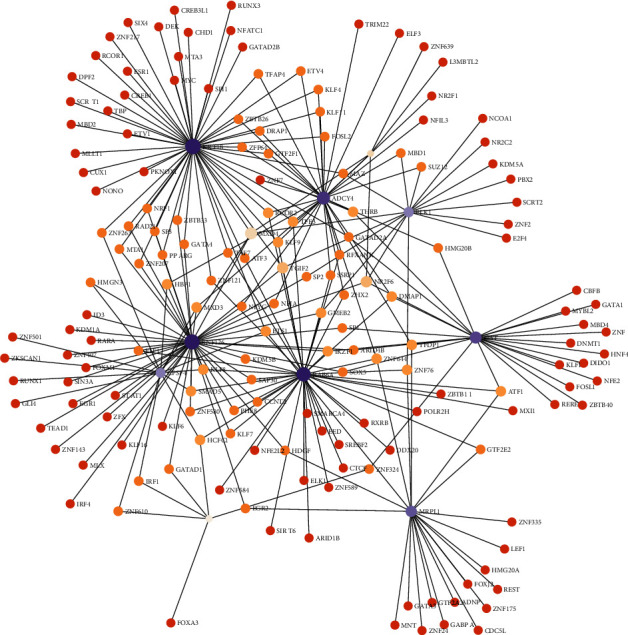
Interaction network of identified genes and transcription factors.

We constructed a regulatory network of 11 key genes and TFs to further explore the potential functions of key genes. TF-gene network included 178 nodes, 263 edges, and 10 seeds, indicating the possible regulatory roles between the hub genes and TFs. Since only subnetworks with at least 3 nodes were chosen to be displayed, the regulatory networks of 10 key genes and 263 TFs are shown in Figure 10 and Supplementary Table 1.

### 3.8. Drug-Gene Interactions

A total of 1985 drugs were screened from the DrugBank, STITCH, CTD, and Broad Institute CMAP Down databases by targeting these 11 genes. We selected the top 30 drugs as candidates according to the FDR value and found that lupenol, thymoquinone, and naringin had potential therapeutic effects on asthma. Surprisingly, the result showed that CAT was the common target gene for all predicted drugs. Lupenol and naringin were mapped with CPSF4 and CAT, while thymoquinone may directly target RAB6A and CAT. The above drugs had the great potential to develop into the therapeutic drugs against SA (Table 1).

### 3.9. ROC Curves for 11 Hub Genes in Samples from Patients with Mild-Moderate Asthma and SA

**Figure 11:**
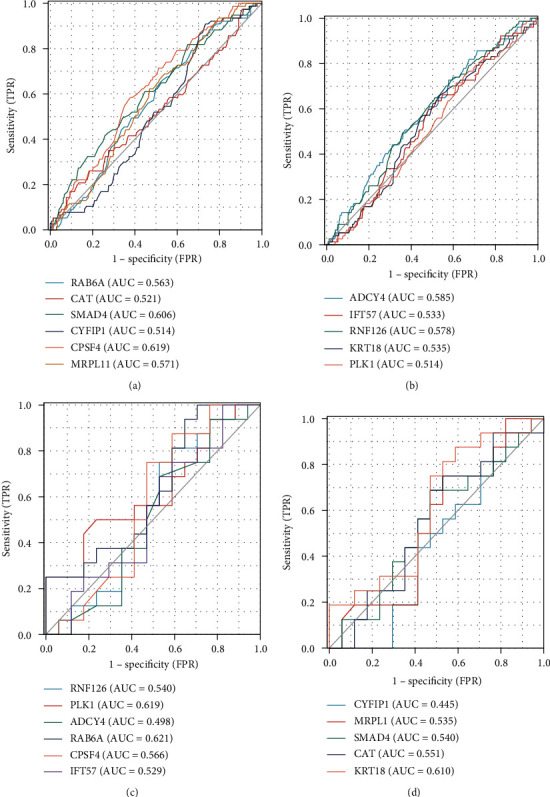
ROC curves of 11 hub genes in the GSE69683 (a, b) and GSE136587 dataset (c, d).

Higher AUC values indicated the more promising diagnostic performance. ROC curves were constructed to assess the diagnostic abilities of 11 hub genes to distinguish moderate asthma from SA in the GSE69683 and GSE136587 datasets. The AUC values (>0.6) for the hub genes in two datasets were listed as follows: SMAD4 (0.606, Figure 11(a)) and CPSF4 (0.619, Figure 11(b)) in the GSE69683 dataset. PLK1 (AUC = 0.619) and KRT18 (AUC = 0.610) had higher AUC values in the GSE136587 dataset. Other genes had AUCs between 0.5 and 0.6 (Figures 11(c) and 11(d)).

## 4. Discussion

SA is a highly heterogeneous disease characterized by uncontrolled symptoms, frequent deterioration, and varying degrees of decreased lung function. Dysregulated inflammation is a key feature of asthma and is regulated by the innate and adaptive immune responses of the immune system [21]. Considering the important role of SA in asthma, the identification of molecular biomarkers and differences in immune cell infiltration between patients with mild-moderate asthma and SA is very important for predicting progression and potential treatments for SA. Therefore, we aimed to use bioinformatics to identify the hub modules strongly related to SA and explore the immune-related mechanisms that may affect the deterioration of SA.

As more than 15 samples are recommended for WGCNA, we obtained a total of 225 SA and non-SA samples from the GSE43696, GSE76226, and GSE89809 datasets to increase the robustness of the results [11]. Next, we normalized the three datasets and evaluated them with PCA analysis. A total of 11105 genes in the three datasets were used to construct 14 coexpression modules with the WGCNA method. We subsequently focused on the pathways and molecular markers of asthma deterioration and determined the correlations of each module with the important clinical factors. We found that the blue module (*P* = 9e − 05) and salmon module (*P* = 6e − 05) had the strongest correlations with SA among the 14 modules. The blue module was positively correlated with SA, and the salmon module was negatively correlated with SA. The analysis of correlations between these two modules and clinical factors further revealed the important value of the blue and salmon modules. Therefore, we continued to analyze the BPs and essential pathways related to these two modules. The blue module was mainly enriched in actomyosin structure organization, muscle cell differentiation, cilium organization, striated muscle cell differentiation, sodium ion transmembrane transport, positive regulation of the catabolic process, and signaling pathways involved in regulating pluripotency of stem cells. The salmon module was mainly related to cornification, skin development, various metabolic processes, glycosphingolipid biosynthesis-lacto and neolacto series, mucin-type o-glycan biosynthesis, and the estrogen signaling pathway. Mesenchymal stem cells (MSCs) can interact with a variety of immune cells, including T lymphocytes, B lymphocytes, and dendritic cells, and exert a strong regulatory effect on the immune system [22–24]. Gao et al. show that human-induced pluripotent stem cell-derived mesenchymal stem cells (iPSC-MSCs) play important roles in the differentiation and function of dendritic cells [25]. As shown in the study by Fang et al., it shows that iPSC-MSCs can regulate Th17 cells, thereby exhibiting the potential to attenuate neutrophil-mediated airway inflammation and inhibit the differentiation of human T helper cells *in vitro* [26]. Keratin, a member of fibrous structural proteins, can protect epithelial cells from stress and damage [27]. Disruption of epithelial barrier function, such as cornification or keratinization, might result in allergic diseases [28]. Inoue et al. find that downregulation of epithelial defense genes and keratinization occurs in SA mouse sensitized by Alternaria [29]. However, Lee et al. demonstrate that keratin KB40 shows an upward trend in asthma mice [30]. Epithelial damage and aberrant repair are present in adult asthmatic airways [31], and there might be potential association between keratinization and epithelial in SA, which needs to be explored and confirmed by more relevant studies. A study has reported that estrogen can enhance the polarization of M2 macrophages induced by IL-4 [32]. Ambhore et al. reveal that estrogen receptors can reduce the deposition of extracellular matrix through the NF-*κ*B signal pathway and can be used as a target to regulate airway smooth muscle remodeling [33].

Going further, PPI and module analyses were conducted to determine the key genes in the blue and salmon modules. We obtained 11 key modules from the blue and salmon modules with the highest degree values, including RNF126, PLK1, ADCY4, RAB6A, CPSF4, IFT57, CYFIP1, MRPL1, SMAD4, CAT, and KRT18. PLK1, a serine/threonine-protein kinase, contributes to reducing airway resistance and airway hyperresponsiveness in asthmatic mice by regulating vimentin phosphorylation at Ser-56 [34]. Phosphorylated SMAD2 and SMAD3 integrate with SMAD4 and are involved in regulating gene transcription in the nucleus [35]. Wortley and Bonvini and Singh et al. suggest that TGF-*β*_1_ attenuates the relaxation of airway smooth muscle induced by *β*_2_ agonists in a SMAD2/3-dependent manner, and the TGF-*β*-SMAD4 axis may be a new therapeutic target for SA [36, 37]. Although the effect of RNF126 on asthma has not been previously reported, it is confirmed that RNF126 plays an oncogene role in a variety of cancers [38, 39]. Xu et al. report that PTEN as an inhibitor of PI3K/AKT signaling pathway can be used as a new substrate of RNF126 [38]. The study reveals that the potential molecular pathways for asthma include FOXC1-miR-PI3K/AKT, which indicates that RNF126 and PI3K/AKT pathways may have therapeutic effect in asthma [40]. Oxidative stress is the main feature of asthma, and genetic variants in the key oxidative defense gene-CAT may have the potential to regulate the risk of new-onset asthma [41]. Targeting oxidative imbalance may help to provide new therapies and researches for asthma treatment [42]. It is well recognized that ADCY4 is also known as AC4. Adenylyl cyclases (ACs) are important regulators of airway smooth muscle function, as *β*-adrenergic receptor (AR) agonists can enhance the activation of AC and increase airway diameter [43]. Bogard et al. assess the levels of AC isoforms in human bronchial smooth muscle cells (hBSMC) and find that AC2, AC4, and AC6 are expressed in hBSMC [44]. High levels of autoantibodies, such as anti-cytokeratin (CK) 18, are found in airway epithelial cells (AECs) [45] and the serum of SA patients [46]. However, the pathogenic mechanism of autoantigens in SA is still poorly understood, which may provide new strategies for the treatment of SA. In addition, there is currently no relevant research disclosing the relationship between RAB6A, IFT57, MRPL1, CYFIP1, CPSF4, and SA, which requires further research in the future.

To further explore the immune infiltration mechanism of SA exacerbation, we used CIBERSORTx to analyze the difference in immune cell infiltration between SA and common asthma and the correlation with hub genes in SA. We found the lower levels of CD8 T cells, T cell follicular helper, and resting mast cells in SA samples than in common asthma samples, but there was more neutrophil infiltration in SA samples. Li et al. find a significant increase in the levels of CD8^+^ T cells in the bronchoalveolar lavage fluid (BALF) of SA patients through single-cell sequencing [47]. Transcriptome analysis conducted by Tsitsiou et al. also shows that SA is associated with circulating CD8(+) T cell activation [48]. Research confirms that activated CD8^+^ T cells show an upward trend in postmortem lung tissue samples of patients who died of acute asthma [49]. TFH cells are a subset of CD4^+^ T cells, which specifically assist the activation and differentiation of B cells to regulate humoral immunity [50]. TFH cells and iconic cytokine IL-21 are related to asthma progression and affect the production of IgE in asthma [51]. Clinical studies have shown that the frequency of TFH cells in peripheral blood mononuclear cells and/or IL-21 levels is positively correlated with IgE levels, which may be promising diagnostic biomarkers for asthma [52]. However, the heterogeneity between patients with moderate asthma and SA still needs further research. Mast cells are present in the skin and most of the mucosal tissues, but the number of mast cells significantly increases in the lungs of asthma patients [53]. And mast cells may play key roles in airway remodeling by releasing tryptase to smooth muscle and epithelial cells [54]. SA often manifests as airway mucosal neutrophil inflammation, and neutrophils can activate inflammatory pathways in AECs through oxidative stress, leading to the exacerbation of neutrophil asthma [7, 55]. More importantly, the formation of neutrophil autophagy and extracellular traps in peripheral neutrophils can increase the severity of asthma by triggering the inflammatory response of the airway epithelium [56]. Taken together, we confirm that our results originated from the analysis of immune infiltration of bronchial epithelial cell transcriptome are not completely consistent with the existing research progress, and further studies need to be carried out to explore the immune infiltration mechanism of SA.

As immune response requires the synergy of immune-related genes and immune cells, we analyzed the correlation between 11 biomarkers of SA and 21 types of immune cells. Macrophages are divided into two types: classical activation (M1) and alternate activation (M2). Studies indicate that the presence of M1 macrophages is associated with disease progression and airway remodeling, and M2 macrophages are associated with type 2 asthma [57, 58]. Tiotiu et al. prove that the number of macrophages in patients with SA significantly reduces compared with mild-moderate asthma patients and healthy volunteers [59]. Takeshita et al. point out that the high expression of PLK1 is significantly related to M0 and M1 macrophages and low levels of M2 macrophages [60]. RAB6A was positively correlated with resting mast cells and negatively correlated with T cell follicular helper and dendritic cells. The resting mast cells and T cell follicular helper in the SA group were significantly lower than those in the non-SA group. RAB6A was the key gene in the blue module, and we speculated that RAB6A can improve the severity of SA via making mast cells quiescent or inhibiting TFH cell expression or reducing dendritic cell levels. However, basic experiments are needed to further confirm. Duvall et al. propose that SA patients show increased proinflammatory granulocytes and CD4^+^ T cells in BALF but decreased NK cells. Corticosteroids can destroy the activity of NK cells leading to NK cell protection deficit, which may be the important mechanism to regulate continuous airway inflammation and dysfunction of SA [61]. Palikhe et al. present that elevated levels of CD4(+) CRTh2(+) T cells are one of the characteristics of SA [62].

TFs can regulate gene expression and are closely related to the occurrence and development of diseases. Notably, SMAD4 was the key target and one of the TFs of ADCY4, indicating that approaches targeting SMAD4 are important in the development of an intervention for SA. PLK1 is a common therapeutic target and overexpressed in a variety of cancers, and targeting PLK1 may enhance the host's innate immune response [63]. However, the molecular mechanism of PLK1 in SA is unclear.

The ToppGene Database makes it possible to screen potential drugs for SA, and we found several potential drugs that may inhibit PLK1. Lupeol, a triterpenoid, can be purified from many plant species and used in popular medicine [64]. Vasconcelos et al. [65] investigate the effect of lupeol in asthmatic mice sensitized by OVA and confirm that lupeol performs potent anti-inflammatory effect by reducing eosinophil numbers in BALF, Th2-associated cytokine levels (IL-4, IL-5, and IL-13), and mucus production with no sign of toxicity. The above research suggests that aiming at lupeol may help to develop new drugs for the treatment of SA. Thymoquinone (TQ, 2-methyl-5-isopropyl-1,4-benzoquinone), a monoterpene molecule present in *Nigella sativa* L., exerts anti-inflammatory and antioxidant effects by releasing cytokines and regulating PI3K/AKT pathway [66]. Surprisingly, it is suggested that TQ decreases the levels of Th2 cytokines in mice with allergic asthma [67]. In addition, TQ suppresses the expression of iNOS and TGF-*β*_1_ mRNAs to prevent inflammatory modification associated with asthma [68]. Naringin can promote the proliferation of AECs by regulating cell cycle progression and activating taste receptor intracellular signaling with no toxic effect to the airway epithelial structure and function [69]. In addition, naringin can reduce the airway resistance of OVA-induced asthmatic mice in a dose-dependent manner to effectively relax murine airway smooth muscle cells *in vitro* and *in vivo* [70]. All of the above researches suggest that naringin is a promising drug for the development of new bronchodilators for SA.

### 4.1. Limitation

In summary, the results of our research are of great significance for understanding the mechanism underlying the infiltration of immune cells in patients with SA based on the transcriptome data of bronchial epithelial cells and multiomics or single-cell sequencing analysis. Second, the results of our research contribute to guiding the further verification in clinical samples, which will promote achievements in the treatment of SA and analyses of the precise mechanisms of key genes and immune cells in SA. However, relevant literature and basic experiments are relatively lacking at present. Studies aiming to further elucidate and verify the mechanism of the hub genes and immune cell infiltration patterns in patients with SA are still necessary.

## 5. Conclusion

This study initially revealed the key genes and immune cell infiltration patterns involved in SA progression. WGCNA showed that the blue and salmon modules were most closely related to SA. RNF126, PLK1, ADCY4, RAB6A, CPSF4, IFT57, CYFIP1, MRPL1, SMAD4, CAT, and KRT18 were crucial genes identified in these two modules. CIBERSORTx algorithms suggested that the differences in CD8^+^ T cells, T cell follicular helper, resting mast cells, and neutrophils between patients with SA and mild-moderate asthma were statistically significant and may be linked to the expression of the 11 key genes. A series of drugs acting on key targets may have strong potential as treatments for SA in the near future. However, more rigorous experiments and verification are needed to be performed to confirm our predicted results.

## Figures and Tables

**Table 1:** Top 30 drug-gene interactions in the severe asthma (according to FDR value).

Category	ID	Name	Source	*P* value	*q*-value FDR B&H	Hit in query list
Drug	CID000000283	Formate	Stitch	0.000006067	0.01473	MRPL1, CPSF4, PLK1, CAT
Drug	CID000102191	V0509	Stitch	0.000006156	0.01473	ADCY4, CYFIP1, IFT57, CAT
Drug	CID000001152	Tungstate	Stitch	0.00001666	0.02193	ADCY4, CPSF4, CAT
Drug	CID005281571	Purpurogallin	Stitch	0.00002875	0.02193	PLK1, CAT
Drug	CID000001184	AC1L1AWW	Stitch	0.00003613	0.02193	KRT18, CAT
Drug	CID000007120	O-108	Stitch	0.00004013	0.02193	KRT18, CAT
Drug	CID000073665	Prochloraz	Stitch	0.00004906	0.02193	ADCY4, PLK1, CAT
Drug	CID000007243	O-phenylenediamine	Stitch	0.000074	0.02193	CPSF4, CAT
Drug	6912_DN	PF-00562151-00 [351320-12-2]; down 200; 10 *μ*M; MCF7; HT_HG-U133A	Broad Institute CMAP Down	0.00008986	0.02193	PLK1, RNF126, CAT
Drug	CID000014195	Benzyl viologen	Stitch	0.0001111	0.02193	CPSF4, CAT
Drug	CID000075038	AC1L2NWK	Stitch	0.0001181	0.02193	KRT18, CAT
Drug	CID000221491	Light yellow	Stitch	0.0001181	0.02193	KRT18, CAT
Drug	CID000160954	Nitrogen oxides	Stitch	0.0001252	0.02193	CPSF4, CAT
Drug	CID000073040	Lupenol	Stitch	0.00014	0.02193	PLK1, CAT
Drug	CID000000321	Coproporphyrinogen III	Stitch	0.0001478	0.02193	CPSF4, CAT
Drug	CID000169577	Extracted from bacteria	Stitch	0.0001557	0.02193	RAB6A, CAT
Drug	CID000010281	Thymoquinone	Stitch	0.0001639	0.02193	PLK1, CAT
Drug	CTD: D002857	Chromium	CTD	0.0001749	0.02193	PLK1, SMAD4, CAT
Drug	CID000000700	Monoethanolamine	Stitch	0.0002077	0.02193	IFT57, CAT
Drug	CID000439504	AC1L97HB	Stitch	0.0002077	0.02193	CPSF4, CAT
Drug	CID000004441	Naringin	Stitch	0.0002171	0.02193	CPSF4, CAT
Drug	CID000005897	2-Acetylaminofluorene	Stitch	0.0002322	0.02193	CPSF4, IFT57, CAT
Drug	CID000077918	1-Benzylimidazole	Stitch	0.0002365	0.02193	CPSF4, CAT
Drug	CID000012446	Dicarbethoxydihydrocollidine	Stitch	0.0002567	0.02193	KRT18, CAT
Drug	CID000001031	Propanol	Stitch	0.0002885	0.02193	ADCY4, CAT
Drug	CID000002612	AC1L1E2H	Stitch	0.0003108	0.02193	ADCY4, CAT
Drug	CID000000866	Dl-lysine	Stitch	0.0003129	0.02193	ADCY4, MRPL1, CAT
Drug	CID000031250	Dltp	Stitch	0.0003222	0.02193	PLK1, CAT
Drug	CID000062638	Tellurium dioxide	Stitch	0.0003338	0.02193	CPSF4, CAT
Drug	CID000007967	Cyclohexanone	Stitch	0.0003338	0.02193	CPSF4, CAT

## Data Availability

The data underlying this article are available in the article and Gene Expression Omnibus public repository.

## Supplementary Materials

Supplementary Materials Supplementary Table 1: identified genes and transcription factors.
