# Supplementary material for: Identification of Molecular Markers Related to Immune Infiltration in Patients with Severe Asthma: A Comprehensive Bioinformatics Analysis Based on the Human Bronchial Epithelial Transcriptome
Source: Dis Markers. 2022 Nov 3;2022:8906064. doi: 10.1155/2022/8906064 (PMC9649321; doi:10.1155/2022/8906064)
Supplement: Supplementary Materials — Supplementary Table 1: identified genes and transcription factors. [file 8906064.f1.docx]

| **Identified genes** | **Transcription factors** |
| --- | --- |
| ADCY4 | SMAD4, NFIA, ETV4, SUZ12, ZNF7, RFXANK, TFAP4, ELF3, NR2F1, DMAP1, FOSL2, KLF9, GTF2F1, DRAP1, MBD1, MXD3, HMG20B, KLF11, THRB, ZNF644, HBP1, ZHX2, KLF4, TFDP1, NFYC, SSRP1, TFE3, TRIM22, RCOR2, ZFP64, NFIL3, ZBTB26 |
| CAT | DMAP1, HMG20B, THRB, TFDP1, NFE2, GATA1, DIDO1, NR2F6, ZNF197, RERE, DNMT1, MYBL2, SP1, ZNF324, IKZF1, CBFB, ZBTB40, KLF1, FOSL1, SOX5, GATAD2A, MBD4, ARID4B, ATF1, HNF4G |
| CPSF4 | MXD3, HBP1, KLF8, ZNF610, HMGN3, SMAD5, ZNF263, ZKSCAN1, GLI4, HCFC1, ZNF501, RUNX1, IRF1, MXD4, ETS1 |
| IFT57 | ZNF324, ZNF610, HCFC1, IRF1, FOXA3, GATAD1 ETV4, TFAP4, FOSL2, KLF9, GTF2F1, DRAP1, KLF11, HRB, KLF4, TFE3, RCOR2, ZFP64, ZBTB26, GATAD2A, ZNF263, MXD4 |
| KRT18 | ETS1, PKNOX1, DEK, NFATC1, ZBTB33, RAD21, PPARG, TAF7, SPI1, TGIF2, CUX1, TBP, ZNF217, CREB3L1, NRF1, MLLT1, ZNF121, CREB1, ATF3, MYC, NONO, DPF2, CHD1, SCRT1, MAZ, MTA1, ETV1, SIX4, GATA4, GATAD2B, ESR1, SP3, MBD2, RUNX3, ZNF207, RCOR1, MTA3 |
| MRPL1 | ZNF644, TFDP1, ATF1, ETS1, MNT, ZNF24, ADNP, CDC5L, HMG20A, GATA3, GTF2A2, ZNF175, LEF1, GABPA, REST, FOXJ2, EGR2, GTF2E2, ZNF76, HDGF, ZNF335 |
| PLK1 | SUZ12, MBD1, NR2F6, MXD4, TGIF2, MAZ, ZNF76, NCOA1, SCRT2, SP2, E2F4, KDM5A, ZNF2, GMEB2, NR2C2, PBX2 |
| RAB6A | RFXANK, DMAP1, KLF9, ZNF644, ZHX2, SSRP1, TFE3, RCOR2, IKZF1, ATF1, KLF8, SMAD5, HCFC1, TAF7, TGIF2, ZNF121, ATF3, GTF2E2, ZNF76, GMEB2, KDM5B, ELF1, ARID1B, ZBTB11, CCNT2, PHF8, SREBF2, SMARCA4, ZNF584, SIRT6, EED, ZNF580, ELK1, NFE2L2, RXRB, POLR2H, SAP30, ZNF589, DDX20, MXI1, KLF7, CTCF |
| RNF126 | NFIA, MXD3, HBP1, NFYC, NR2F6, SP1, IKZF1, SOX5, ARID4B, KLF8, HMGN3, SMAD5, MXD4, GATAD1, ZBTB33, RAD21, PPARG, TGIF2, NRF1, MTA1, GATA4, SP3, ZNF207, EGR2, HDGF, SP2, GMEB2, KDM5B ELF1, CCNT2, PHF8, ZNF580, SAP30, KLF7, ZNF143, ID3, MLX, RARA, SIN3A, STAT1, KLF6, TEAD1, FOXM1, ZFX, KLF16, IRF4, ZNF407, KDM1A, EGR1 |
| SMAD4 | NR2F6, MXD4, ZNF639, L3MBTL2 |

**Supplementary Table 1: Identified genes and transcription factors.**
